# Semi supervised data mining model for the prognosis of pre-diabetic conditions in type 2 Diabetes Mellitus

**DOI:** 10.6026/97320630015875

**Published:** 2019-12-31

**Authors:** A Sumathi, S Meganathan

**Affiliations:** 1 Department of Computer Science Engineering, SASTRA Deemed To be University, Srinivasa Ramanujan Centre (SRC), Kumbakonam, Tamil Nadu, India; 2 Department of Computer Science Engineering, SASTRA Deemed To be University, Srinivasa Ramanujan Centre (SRC), Kumbakonam, Tamil Nadu, India

**Keywords:** Diabetes, semi supervised learning, epidemiological data, prognosis, classification, clustering

## Abstract

Diabetic Mellitus is the leading disease in the world irrespective of age and geographical location. It is estimated that 43% of the overall population is affected by the disease. The reasons for the disease include inappropriate diet lifestyle with allied symptoms like obesity. Therefore, the prognosis and diagnosis of the disease are important for adequate combat and care. The prognosis related known symptoms of the disease include incontinence (inability to control urination) and frequent fatigue. Moreover, early prediction of the disease plays an important role in the prognosis of other associated conditions such as heart failure leading to chronic illness. Hence, it is of interest to describe a data mining based prediction model using known features (derived from epidemiological data collected from the public hospital using routine tests) to help in the prognosis of the disease. We used data pre-processing techniques for handling missing values and dimensionality reduction models to improve data quality. The Minimum Description Length principle (MDL) model for discretization (replacing a continuum with a finite set of points) is used to reduce high-level dimensionality of the dataset, which enabled to categorize the dataset into small groups in ordered intervals. Thus, we describe a semi-supervised learning technique (identifies promising attributes using clustering and classification methods) by combining data mining techniques for reasonable accuracy having adequate sensitivity and specificity for further discussion, cross-validation, revaluation, and application. Early prediction of the disease with improved accuracy by analysing specificity ranges in blood pressure and glucose levels will be useful to combat Diabetes Mellitus.

## Background

**Figure 1:**
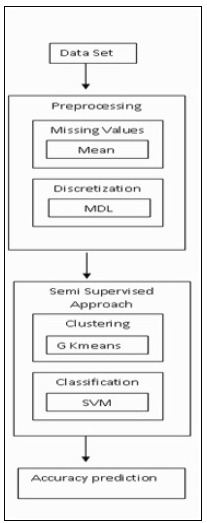
Semi supervised learning model for predicting pre-diabetes occurrences

The world has become health conscious. People are interested in understanding the root cause of diseases. Most diseases are caused in two major ways. The first cause is uncontrolled food intake and other cause is due to alcohol and tobacco consumption. The disproportionate intake of unhealthy food is linked with diseases like cancer, obesity, etc. However, inadequate intake of food causes anemia, mineral deficiency, mal nutrition and other diseases. Diabetes Mellitus [1] is one of the life-threatening diseases, which causes due to dysfunction of the pancreas due to insufficient secretion of insulin irrespective of age. Diabetic Mellitus is a silent killer [2]. The known subsidiary diseases of diabetes are retinopathy, heart disease, and chronic kidney disease. Glucose test before and after food intake is common [3,4]. Data Mining [5] is a powerful technique used to identify the hidden patterns from data repositories. The prognosis of pre diabetes symptoms using known epidemiological data is useful in treatment. The standard data mining algorithms are used to identify, analyze the hidden data patterns and collect relationships in known stored data. The building of interconnected relations in known clinical data shows different combinations of symptoms towards combat and care. Therefore, it of interest to use machine learning [5,6] and especially Semi-supervised learning [7,8] exploiting known classification and clustering techniques to help in the prognosis diabetes (Figure 1).

## Data Mining in Biological domain:

Data Mining is one of the oldest and standard techniques for handling biological data in different forms [8,9]. The inherent nature of the complexity of biological data requires a collection of techniques to analyze the data from large datasets. The information retrieval is possible using data mining with contextual results relating multiple dimensions. The use of Machine learning for data mining in large data is becoming common. Further, the use of the semi-supervised learning [9] in analysing both labelled and unlabeled data is getting familiar. Thus, the use of machine learning especially semi-supervised learning approaches in biological data mining finds immense application in biological knowledge discovery [10].

## Methodology

Data mining techniques are used for medical applications. The single approach always gives results for a particular approach with less optimal output. A combination of approaches is needed to handle a different set of attributes with interrelationships. Data pre-processing [11] is primary in data mining. The use of MDL (Minimum Description Length) in diabetes linked data mining is known. Our interest is to use machine learning especially semi-supervised learning approaches in diabetes linked data mining to glean useful information related to the disease.

## Preprocessing:

Data preprocessing [12] is a necessary to remove noise to improve accuracy. The calculation of the average mean value in a given dataset is primary in this context. The formula used for mean is given below:

The Mean (x) = (sum) of all the values Σ(x) / the number of values (n).

mean(x) = Σ(x)/n

The x represents the inputs of patient attribute values.

## Discretization:

Biological data is multidimensional. Hence, minimization of its dimensionality is the first step for categorization. The discretization [13] minimizes the distance between related values of attributes by ordering it. The ordered set is well organized with minimal and maximum intervals. It reduces the large chunks of numeric values into a group of well-organized values. The discretization contains many techniques. MDL is a suitable method for identifying the most promising attribute.

## Minimum Description Length (MDL):

MDL is based on information theory and entropy using data representation to find regular patterns in data [14]. MDL helps to filter the most relevant values in the normal values. We used the following entropy formula in MDL discretization.

Entropy(D1) = -Smi=1pilog2pi

## Clustering and Genetic k-means algorithm:

**Figure 2:**
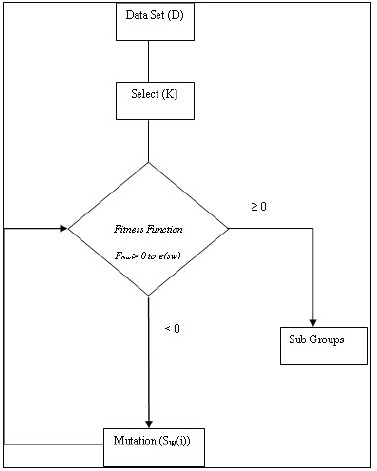
Genetic k-means algorithm for Type 2 diabetes mellitus prediction

The Clustering technique is a simple and powerful technique that is used to categorize the unlabelled data into groups called clusters. Similar and relevant data points are organized into groups during clustering. It is an iterative technique, which uses updated centerdata point in each iteration. The limitation in clustering is its local maximum. The group needs a generalized scope to increase its scope in a cluster to achieve better scope. Global maximum is needed for generalized applications. The general k-means clustering algorithm with a genetic approach is combined to form a genetic k means algorithm to predict pre diabetic conditions. The genetic k means [15] is an evolutionary approach that gives optimum solutions for complex problems to achieve local maximum with good performance in a diabetic dataset (Figure 2).

The fitness function is applied for clustering is given below.

P(Si) = F(Si)/SNj=1F(Sj)

## Classification using Support Vector Machine (SVM):

**Figure 3:**
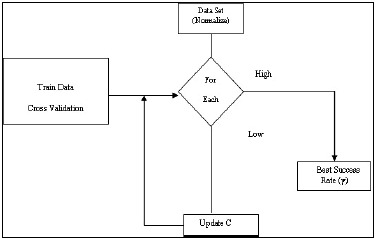
SVM algorithm for possible occurrence of Type 2 diabetes mellitus

SVM helps to distinguish data classes and concepts. Classification [16,17] helps in identifying known data from unknown resources. The known data is called training data and the unknown data is called test data. In a semi-supervised learning approach, the Diabetic data set attributes and ranges are categorized using the clustering genetic k means method. Therefore, the set of categories (subpopulations) and new observation belongs to the basis of a training set of data containing observations and whose categories are known. The SVM [17] is a training algorithm, which builds a model that assigns new examples to one category, by making it a non-probabilistic binary linear classifier. The SVM performs well compared with other statistical or machine learning methods, especially with biological datasets. Kernel methods like the SVM can easily handle non-vector inputs unlike most machine learning methods. It evaluates prognostic models by separating the initial information set as a coaching data. Thus, the SVM classification rule provides higher accuracy in the result (Figure 3).

## Dataset:

The dataset consists of 2106 person records collected from a Government Hospital, Kumbakonam, Tamil Nadu, India. The record consists of basic health checkup of an individual with 11 attributes that are all numeric. The attribute contains the habitual activities of a person, blood pressure, height, weight, BMI, blood glucose level, age, gender etc.

## Results and Discussion:

**Figure 4:**
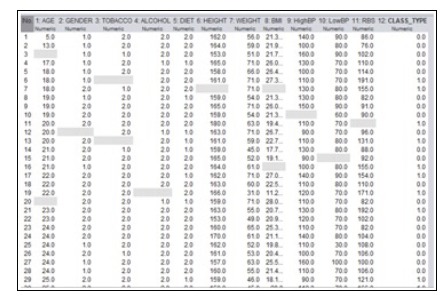
Dataset with missing values

**Figure 5:**
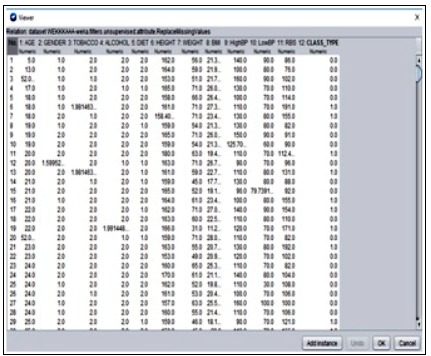
Replacing of missing values using mean values

**Figure 6:**
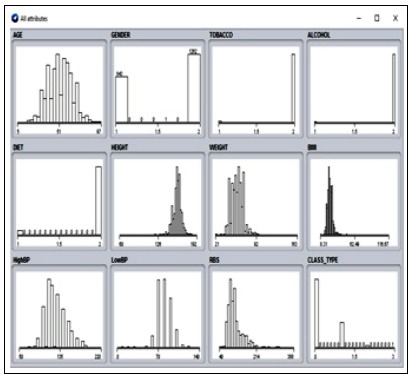
Dataset before discretization

**Figure 7:**
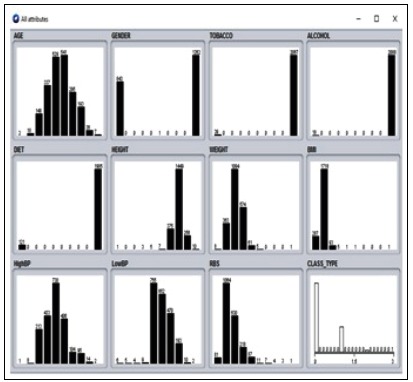
After discretization using MDL techniques with ordered attributes

**Figure 8:**
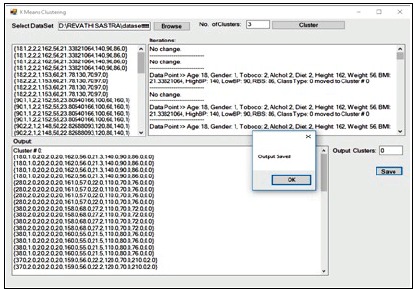
Clustered Type 2 diabetes mellitus data using genetic k-means algorithm

**Figure 9:**
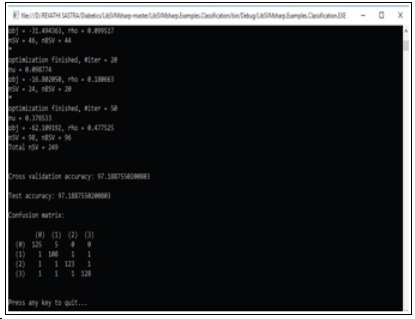
SVM based classification for Type 2 diabetes mellitus data

The described model consists of three steps: (a) Data pre-processing; (b) Clustering and (c) Classification. The preprocessed data is then clustered using the genetic k-means technique producing labeled categorized data. The labeled data is then classified into subgroups based on the attribute intervals by using the SVM technique. The possible diabetic occurrence promising attribute values are identified using sub categorization done by SVM classification. The analysis is done in the WEKA [18] tool where the real-time obtained data is given as input. The missing values in the given data set are removed by replacing the average mean of an attribute. The data is loaded into the WEKA tool and missing values are identified (Figure 4 and Figure 5). The missing values are replaced using the mean average replacement technique during preprocessing. The mean minimizes the error ratio with improved accuracy. The values for attributes are ordered. The dimensionality of attributes is reduced in the category. The preprocessed data is then analyzed using the MDL technique (Figure 6). The output of MDL implementation gives the ordered values of attributes (Figure 7). The second step of the model involves the labeling of data using Genetic k-means clustering. The preprocessed data set using mean and MDL are given as input. The initial cluster k value is given as 3 and the data set is divided into three labeled clusters whereas it contains sub-clusters with the nearest ranges (Figure 8). The third step is classification to identify the normal values in the range. The SVM uses correlation and regression to build relationships among the given attributes. Therefore, the sub-categorization of labeled data enables to predict promising attribute values of Type 2 diabetes mellitus occurrences. The performance of the SVM classifier was evaluated using the confusion matrix. The classifier results are shown in (Figure 9). Analysis shows results from the preprocessing implementation using mean values in MDL. The semi-supervised learning approach is applied as a combined technique of genetic k-means and SVM classifiers in this study. The unlabelled data is categorized using the genetic k-means algorithm with optimal subgroups as clusters. The data points in each attributed values are updated during iteration for every biomarker. Each attribute values are clustered as a subgroup with nearby data points. The SVM classifier is used to categorize the relevant attributes as sub-groups. The classifier relates the attribute values and separates the promising attribute values from the normal range attribute values. The promising attributes values are the possible values of the occurrence of the disease. The most significant value in each attribute is correlated with the next promising attribute value to improve the accuracy rate in diabetic prognosis.

## Conclusion

We describe a semi-supervised knowledge discovery model by finding attributes using clustering and classification procedures in data mining for reasonable precision with sufficient accuracy for further debate, confirmation, revaluation and application in the prognosis of pre-diabetic conditions for Type 2 diabetes mellitus. We used the data collected from a public hospital for this analysis. The dataset is used as training set for the model. The model identified the promising attributes using clustering and classification techniques. The data preprocessing techniques for handling missing values and dimensionality reductions are used to improve the quality of data. The MDL discretization technique reduced the high-level dimensionality of the dataset. This enabled to categorize the dataset into small groups in ordered intervals for improved accuracy.

## References

[1] Tse SY (2018). BMC FamPract.

[2] Wanner C, Marx N (2018). Diabetologia.

[3] Ikwuobe J (2016). Biogerontology ..

[4] Kirk IK (2019). Elife.

[5] Kraniotou C (2018). J Proteomics..

[6] Kavakiotis I (2017). Comput Struct Biotechnol.

[7] Dagliati A (2018). J. Diabetes Sci Technol..

[8] Chapelle O (2006). Semi-Supervised Learning.

[9] Woldaregay AZ (2019). ArtifIntell Med..

[10] Dong-giLee J (2018). Expert Systems with Applications ..

[11] Nie F (2018). IEEE Trans Image Process..

[12] Bakariya B (2012). Advances in Intelligent Systems and Computing.

[13] Zhu M (2011). Communication Systems and Information Technology.

[14] Quinlan JR (1986). Machine Learning.

[15] Dhillon PS (2011). Machine Learning Research.

[16] Krishna K (1999). IEEE Transactions on Systems, Man, and Cybernetics, Part B.

[17] Miroslav Marinov (2011). J Diabetes Sci Technol..

[18] Cui S (2018). Comput Methods Programs Biomed..

[19] Smith TC (2016). Methods Mol Biol..

